# Aged Oolong Tea Reduces High-Fat Diet-Induced Fat Accumulation and Dyslipidemia by Regulating the AMPK/ACC Signaling Pathway

**DOI:** 10.3390/nu10020187

**Published:** 2018-02-08

**Authors:** Erdong Yuan, Xuefei Duan, Limin Xiang, Jiaoyan Ren, Xingfei Lai, Qiuhua Li, Lingli Sun, Shili Sun

**Affiliations:** 1 School of Food Science and Engineering, South China University of Technology, Guangzhou 510641, China; erdyuan@scut.edu.cn (E.Y.); dxf_artemis@126.com (X.D.); jyren@scut.edu.cn (J.R.); 2 Tea Research Institute, Guangdong Academy of Agricultural Sciences, Guangdong Provincial Key Laboratory of Tea Plant Resources Innovation & Utilization, Guangzhou 510640, China; xianglimin@tea.gdaas.cn (L.X.); laixingfei@tea.gdaas.cn (X.L.); liqiuhua@tea.gdaas.cn (Q.L.); 3 Sino-Singapore International Joint Research Institution, Guangzhou Knowledge City, Guangzhou 510000, China

**Keywords:** anti-obesity, anti-inflammation, water extract, aged oolong tea

## Abstract

While oolong tea (OT) has been shown to induce weight loss and reduce fat accumulation, the mechanisms remain poorly defined, especially for aged OT. In this study, five groups of mice (*n* = 9/group) were used including a normal diet with vehicle treatment, and a high-fat diet (HFD) with vehicle or the water extracts from aged OTs (EAOTs, three different storage years) by oral gavage at 1000 mg/kg·BW for 6 weeks. Body weight, fat accumulation, and serum biochemical parameters were used to evaluate obesity. The morphology of hepatocytes and adipocytes was analyzed by being stained with hematoxylin and eosin. The levels of p-AMPK, p-ACC (and non-phosphorylated versions), CPT-1 and FAS were determined by Western blotting and immunohistochemistry. EAOTs decreased HFD-induced body weight, fat accumulation, serum levels of triglyceride, total cholesterol, and low-density lipoprotein cholesterol, while enhancing the serum high-density lipoprotein cholesterol level. At the same time, EAOTs clearly alleviated fatty liver and reduced the size of adipocytes in the epididymal fat, especially in the 2006 group. Most importantly, EAOTs increased the phosphorylation of AMPK and ACC, and up-regulated the expression of CPT-1 but down-regulated the expression of fatty acid synthase, TNF-α and iNOS. Thus, EAOTs may inhibit obesity by up-regulating energy expenditure and fatty acid oxidation while inhibiting fatty acid synthesis and inflammation.

## 1. Introduction

High energy intake, malnourished diets containing lots of fat, and refined carbohydrates coupled with a sedentary lifestyle are believed to contribute to the global obesity epidemic [1,2]. Obesity tends to result in other pathological disorders, such as diabetes, atherosclerosis, hypertension, and cancer [3,4,5]. It is estimated that the prevalence of obesity will increase by 7% and 10% among men and women, respectively, by 2020 [6]. As one of the most popular beverages consumed worldwide, tea has been shown in numerous clinical trials to affect body weight and fat metabolism in humans [7] and in rodents [8] fed high-fat diets (HFDs). Oolong tea (OT) is a kind of partially fermented Chinese tea, and it is considered to have a stronger weight loss effect than any other kinds of tea. Previous research has shown that OT leaves can remarkably decrease rats’ body weight [9]. However, the effects of the water extract from aged OTs (EAOTs, three different storage years) on fat deposition and inflammation, as well as potential mechanisms of action, were not adequately studied. During storage, the contents of tea polyphenols, soluble sugar, and other ingredients changed [10]. As these ingredients are the material basis of OT’s anti-obesity and anti-inflammation effects, it is reasonable to speculate that the physiological effects of OT would change with storage as well.

The aim of this study was to investigate the effects of EAOTs on anti-obesity and anti-inflammation. Additionally, we analyzed the protein expression following EAOT treatments to evaluate HFD-induced pathways of alleviating obesity and inflammation. Therefore, the present study could demonstrate new insights into the anti-obesity and anti-inflammation mechanism induced by EAOTs.

## 2. Materials and Methods

### 2.1. Materials

Dancong Oolong tea (a kind of OT, stored in 2016, 2006, 1996 separately) was purchased from Dapuxiyan Tea Group Co. Ltd. in Meizhou, Guangdong, China.

### 2.2. Preparation of EAOTs

Powder prepared from OT was extracted three times by placing in boiling distilled water for 30 min each time (tea/water, 1:20 *w*:*v*). These extracted solutions were combined and centrifuged, then concentrated at 60 °C. Finally, the solution was dried by lyophilization.

### 2.3. Determination of Ingredients in EAOTs

The water content was measured by comparing the weight difference between before and after heating in a 130 °C oven for 3 h, and the content of free amino acids was determined by the ninhydrin method. In addition, anthrone–sulfuric acid colorimetric assay was used to determine the total soluble sugar content, and the Folin-phenol method for tea polyphenols.

### 2.4. Animals

Male, 7-week-old C57BL/6J mice were purchased from Beijing Huafukang Bioscience Co. Ltd. (Beijing, China). All experimental procedures were conducted in accordance with institutional guidelines for the care and use of laboratory animals, and all efforts were made to minimize animal suffering. The protocols were approved by the Ethical Committee of Tea Research Institute (2015-005) at Guangdong Academy of Agricultural Sciences.

Mice were individually housed in standardized conditions for animal facilities: a 12-h light/dark cycle (off at 7 pm), 23 ± 2 °C room temperature, and 55 ± 5% relative humidity. Mice had ad libitum access to water and food (Beijing Huafukang Bioscience Co., Ltd.).

### 2.5. Diet-Induced Obesity

After a week of adaptation, 45 C57BL/6J were randomly divided into five groups as follows: (i) the control group (*n* = 9); (ii) the group fed an HFD (Model) (*n* = 9); (iii) the group fed an HFD and 1000 mg/kg·BW EAOTs stored in 2016 (2016) (*n* = 9); (iv) mice fed an HFD and 1000 mg/kg·BW EAOTs stored in 2006 (2006) (*n* = 9); and (v) the group fed an HFD and 1000 mg/kg·BW EAOTs stored in 1996 (1996) (*n* = 9). Control mice were fed a normal diet (containing protein 18%, fat 4%, carbohydrate 62%, fiber 5%, minerals 8%, and vitamins 3%, *w*:*w*; Beijing Huafukang Bioscience Co., Ltd., 1022), and mice in the other four groups were fed an HFD (45% calories from fat, 20% calories from protein, 35% calories from carbohydrate; Beijing Huafukang Bioscience Co., Ltd., H10045) for 18 weeks, during which time all mice were not administered EAOTs.

### 2.6. EAOT Treatment

After 18 weeks, the mice in all groups remained on the same diet as before. Mice from three EAOT treatment groups were given, by gavage, 1000 mg/kg·BW EAOTs daily for approximately 6 weeks, while the control group and the model group mice were given, by gavage, distilled water at the same time. Body weight, food, and water intake were recorded once a week. At the end of the experimental period, the mice fasted for 12 h, and then blood samples were collected by cardiac puncture technique under carbon dioxide anesthesia. Thereafter, the mice were euthanized with an anesthetic overdose, and were perfused transcardially through the ascending aorta with normal saline so as to remove any blood clots from tissues and organs. The liver was rapidly removed, weighed, and washed thoroughly with phosphate buffer saline (PBS, pH 7.4). Half hepatic lobar was preserved in 10% buffered formalin solution for histopathological examinations. The rest of the liver was homogenized in ice-cold phosphate buffer saline, then stored in liquid nitrogen for various biochemical and molecular assays. The entire procedure was carried out under cold conditions.

### 2.7. Lee’s Index

Lee’s index is similar to the human body mass index, and mainly reflects the proportion of body fat in the total weight. It can characterize the degree of obesity more accurately than body weight [11]. The smaller the Lee’s index, the more slender the body. After the animals were sacrificed, their body weight and length were measured to calculate the Lee’s index.

Lee’s = [weight (g)/length (cm)]^1/3^

### 2.8. Serum Chemistry Analysis

After being kept at room temperature for 30 min, blood samples were centrifuged at 13,200 r/min for 20 min at 4 °C, and subsequently stored at −80 °C. The serum was used to measure triglycerides (TGs), total cholesterol (TC), high-density lipoprotein cholesterol (HDL-C), and low-density lipoprotein cholesterol (LDL-C) levels (iMagic-V7 Automatic Analyzer, ICUBIO, Beijing, China).

### 2.9. Histological Examination

The liver and epididymal fat sections fixed in 10% neutral buffered formalin were dehydrated in graded concentrations of alcohols, and embedded in paraffin. Then the tissue was sectioned at 4 μm and stained with hematoxylin and eosin (H&E). The pathological changes were assessed and photographed on an Olympus BX-53 microscope (Olympus, Tokyo, Japan).

### 2.10. Western Blotting Analysis

Protein from each liver sample was extracted with lysis buffer at 4 °C. Then, the extracts were centrifuged at 13,200 r/min and 4 °C for 30 min, and the supernatants of these tissues were used for Western blotting analyses. The protein samples were separated on 10% SDS-PAGE gels and electrophoretically transferred onto PVDF membranes. The membranes were blocked at room temperature with 5% non-fat dry milk in TBST for 1 h, and then incubated overnight at 4 °C with the indicated primary antibodies as follows: AMP-activated protein kinase α (AMPKα), p-AMPKα (Thr172), acetyl-CoA carboxylase (ACC), p-ACC (Ser79), camitinepalmitoyl transferase 1 (CPT-1), fatty acid synthase (FAS), iNOS (Cell Signaling Technology, Danvers, MA, USA), TNF-α (Abcam, Cambridge, MA, USA), and β-actin (Sigma-Aldrich, St. Louis, MO, USA). After washing three times with TBST, the blots were hybridized with secondary antibodies conjugated to horseradish peroxidase. The proteins were visualized by enhanced chemiluminescence.

### 2.11. Immunohistochemistry (IHC)

The liver paraffin samples were sliced 4 μm thick and were dewaxed in xylene and rehydrated through a graded series of ethanol concentrations. Then, the slides were pretreated with a microwave antigen retrieval technique and blocked with 5% BSA. The slices were incubated overnight at 4 °C with diluted primary antibodies (Cell Signaling Technology, Danvers, MA, USA). After three washes with PBS for 5 min, the sections were incubated with secondary antibodies (Boster, Wuhan, China) for 30 min at 37 °C. Horseradish peroxidase-conjugated anti-rabbit IgG and anti-mouse IgG (Beyotime, Shanghai, China) were used for enhanced chromogenic reaction. After washing, the DAB chromogen was added for 3 min before counterstaining with hematoxylin. Images of immunostained liver tissues were taken with an Olympus BX-53 microscope (Olympus, Tokyo, Japan).

### 2.12. Statistical Analysis

Repeated measures analysis of variance (ANOVA) was conducted, and Tukey’s post-hoc test was used to adjust for multiple comparisons. A *p*-value < 0.05 was considered statistically significant, and Prism 6.0 software for Windows (GraphPad Software, La Jolla, CA, USA) was used to conduct statistical analyses. Data are presented as mean ± standard error of the mean (SEM) for effects of EAOT treatment on body weight, food intake, water consumption, and Lee’s index, and mean ± standard deviation (SD) for all other results.

## 3. Results

### 3.1. The Ingredients of EAOTs

As shown in Table 1, the free amino acids content was significantly lower in aged oolong tea, because of the degradation and polymerization during the storage time. Meanwhile, microorganisms may also have consumed protein and decreased this content.

The content of total soluble sugar markedly increased with the storage time, as macromolecular carbohydrates gradually decomposed when stored.

However, with the storage time, the change in tea polyphenols content was nonlinear. The 2006 group had a higher tea polyphenol content compared to the other two groups. Similar results were also seen in other studies, although for a shorter period [12].

### 3.2. Body Weight, Food and Water Intake, and Lee’s Index

**Figure 1:**
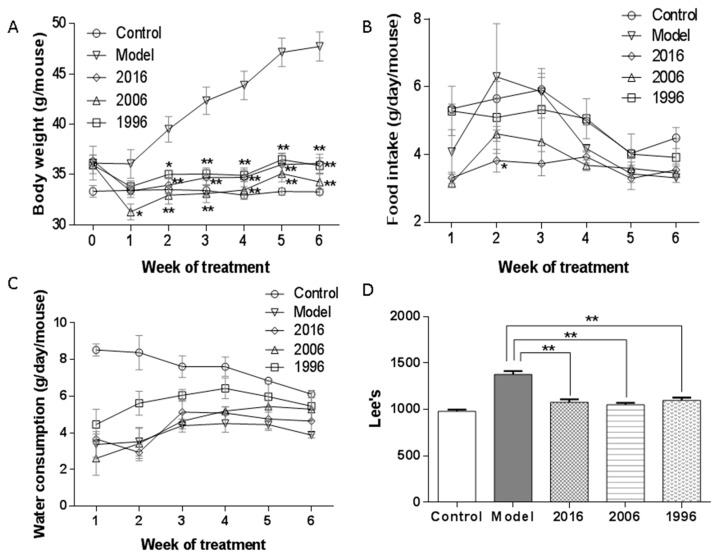
Effects of EAOT treatment on (**A**) body weight; (**B**) food intake; (**C**) water consumption; and (**D**) Lee’s index. During 6 weeks of different storage years of EAOT treatment, body weights, food intake, and water consumption were recorded once a week. After 6 weeks of administration, Lee’s index was measured. Data were mean ± SEM (*n* = 9). * *p* < 0.05 versus model; ** *p* < 0.01 versus model.

The effect of EAOTs on obesity was investigated using male C57BL/6J mice with HFD-induced obesity. Although the average body weight did not significantly differ between the model group and three treatment groups at week 0, the latter had significantly lower body weight than the model group, and had even reached the level similar to the control group from the second week to the end of the trial (Figure 1A). The 2006 group showed slightly lower body weight than the 2016 and 1996 groups, but the difference was not statistically significant. However, no marked differences were observed in daily food and water intake among all groups, showing that EAOTs did not suppress food and water intake (Figure 1B,C). At the end of the animal test, Lee’s index was also measured to evaluate the degree of mice obesity. In the groups fed the HFD and EAOTs, Lee’s indexes were significantly prevented compared to that of the mice fed only the HFD (Figure 1D).

### 3.3. EAOTs Attenuate Fatty Liver and Adiposity in HFD-Induced Obese Mice

**Figure 2:**
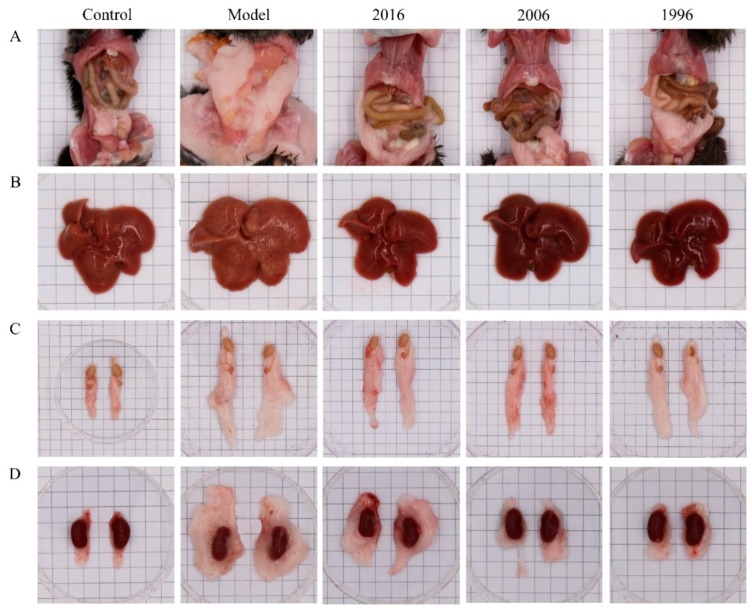
EAOTs attenuate fatty liver and adiposity in high-fat diet (HFD)-induced obese mice. Representative images of (**A**) whole body; (**B**) liver; (**C**) epididymal fat; (**D**) and pararenal fat in all groups.

The effects of EAOTs on fat accumulation were studied by anatomic observation and by analyzing the organ index of white adipose tissues. Gross observation revealed that the mice in the model group showed a large accumulation of white fat, while it decreased markedly after being treated with EAOTs, especially in the 2006 group (Figure 2A). In Figure 2B, the model group mice had developed fatty livers. Their liver sizes were larger than those of the control group. They also became yellow-brown and the liver surfaces were uneven, whereas the liver changes of mice treated with EAOTs were equal to those of the control mice. Moreover, EAOTs greatly decreased the size of epididymal and pararenal adipose tissue masses (Figure 2C,D).

At the end of the animal experiment, the body weight, organ weight, and adipose tissue weight of the mice were measured. As shown in Table 2, the hepatic indexes were evidently diminished in EAOT-treated obese animals, as well as the fat indexes. Moreover, the intestinal fat indexes of the mice in the EAOT treatment groups were sharply decreased to a level similar to those in the control group, and the pararenal fat indexes in the 2006 group mice showed no significant differences with those of the mice in the control group. As a whole, the administration of EAOTs inhibited the increase of the white fat index, particularly in the 2006 group. In contrast, the model group mice had a markedly reduced kidney index, comparable to that seen in the control group. After EAOT treatment, this index increased to a level similar to that of the control mice (Table 2). However, there were no significant differences between each group in the sizes of kidney (Figure 2D), implying that this drop in model group was mainly because of the increase in body weight.

### 3.4. Effects of EAOTs on Serum Levels in HFD-Induced Obesity Mice

Obese people usually suffer from hyperlipidemia, so the serum biochemistry was evaluated to assess the anti-obesity effects of EAOTs in HFD mice. The model group had the highest TG, TC, and LDL-C, and the HDL-C level was lowest in the model group (Table 3). EAOT treatments resulted in a dramatic decrease in TG, TC, and LDL-C, and a notable increase of HDL-C, which were similar to those of the control mice. Moreover, a moderately lower concentration of TG, TC, and LDL-C was found in the 2006 group than in the 2016 and 1996 groups (Table 3). Together, these results clearly revealed that EAOTs could inhibit hyperlipidemia in obese animals.

### 3.5. Effect of EAOTs on Accumulation of Lipid Droplets in Liver and Epididymal Fat

**Figure 3:**
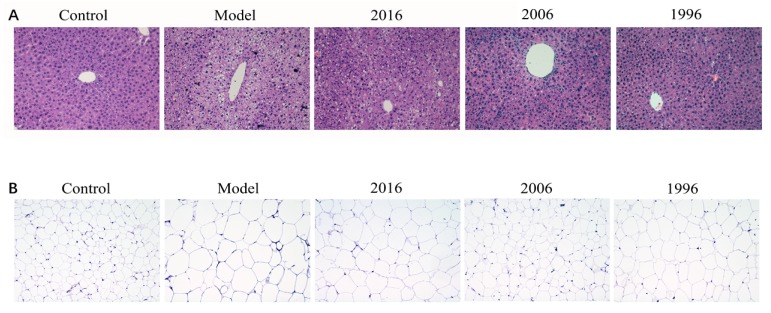
Effect of EAOTs on accumulation of lipid droplets in liver and epididymal fat. Representative hematoxylin and eosin (H&E) staining images of (**A**) liver and (**B**) epididymal adipose tissue (scale bar 20 μm).

To determine whether the observed decrease in liver weight was due to a reduced accumulation of fat, we stained representative liver with H&E. As shown in Figure 3, the model group mice showed an increase in lipid accumulation and pronounced steatosis in the liver, which was characterized by prominent ballooning injury, vacuolation, and hepatocellular hypertrophy. EAOTs clearly alleviated fatty liver, and lipid deposition was markedly decreased, especially in the 2006 group (Figure 3A).

For adult mice, the effect of obesity on adipocyte is mainly the increase of cell volume. We also examined the epididymal adipose tissue histology of obese mice in the absence and presence of EAOTs. Enlargement of adipocytes in the epididymal fat of mice in the model group was clear, and it was revealed that the size of adipocytes in the epididymal fat was definitely suppressed in the EAOT treatment groups compared with the model group. Moreover, the diameter of lipid droplets in the 2006 group was similar to that in the control group (Figure 3B).

### 3.6. EAOTs Activate AMPK and ACC Phosphorylation

**Figure 4:**
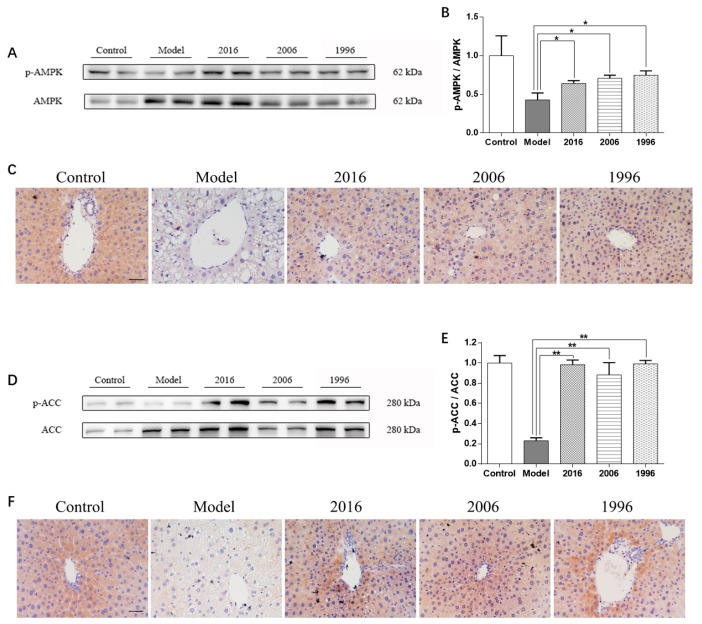
EAOTs activate AMPK and ACC phosphorylation. (**A**) Western blotting analysis of AMPK protein levels in mice liver and (**B**) densitometric quantification of them (*n* = 3 independent experiments); (**C**) Immunohistochemical staining of liver for p-AMPK; (**D**) Western blotting analysis of ACC protein levels in mice liver and (**E**) densitometric quantification of them (*n* = 3 independent experiments); (**F**) Immunohistochemical staining of liver for p-ACC. The scale bar represents 20 μm. Each value represents the mean ± SD (*n* = 9); * *p* < 0.05 versus model; ** *p* < 0.01 versus model.

AMPK is able to dissipate excessively stored energy by stimulating fatty acid oxidation. To investigate the effect of EAOTs on AMPK activation in mice liver, immunoblot analysis was performed. AMPK activation was determined by measuring the phosphorylation level of the AMPKα subunit at Thr172, which reflects the activation of AMPK [13]. In the liver tissue of obese mice, the HFD led to a radical decrease in the phosphorylation degree of AMPKα compared to the control group, while EAOT treatments resulted in exponentially increased AMPKα (Thr172) phosphorylation (Figure 4A). Moreover, the p-AMPKα/AMPK ratio decreased by 57.1% in the model group mice livers, whereas it increased in the mice fed the HFD and EAOTs (Figure 4B). In IHC, brown signals were mainly confined to p-AMPKα. The IHC signals were more intense and a significantly higher number of positive protein was observed in the livers of the mice in the treatment groups than that of the model mice, which showed mild signals and a lower number of positive protein (Figure 4C).

ACC activation was determined by measuring the phosphorylation level of ACC at Ser79 where AMPK phosphorylates [14]. Similarly, the phosphorylation degree of ACC (Ser79) in the livers of the mice fed with the HFD was down-regulated compared with those in the control group. Furthermore, the HFD significantly reduced the p-ACC/ACC ratio (by 77.0%) in the livers of the mice. These HFD-induced down-regulations in the phosphorylation degree of ACC (Ser79) and the ratio of the p-ACC/ACC were markedly reversed in the livers of the mice in the treatment groups (Figure 4D,E). The phosphorylation of ACC (Ser79) was also assessed by IHC. The density of p-ACC (Ser79)-positive cells was radically increased in EAOT-treated mice compared to the model group (Figure 4F).

### 3.7. EAOTs Activate CPT-1 Expression and Inhibit FAS Expression

**Figure 5:**
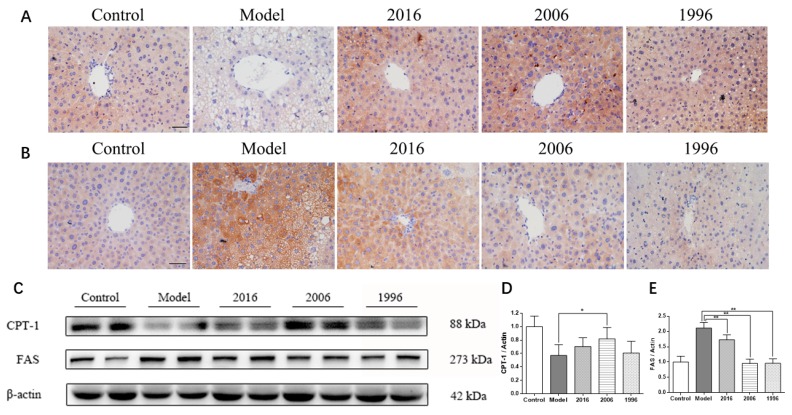
EAOTs activate CPT-1 expression and inhibit FAS expression. Immunohistochemical staining of liver for (**A**) CPT-1 and (**B**) FAS; (**C**) Western blotting analysis of CPT-1 and FAS protein levels in mice liver and densitometric quantification of (**D**) CPT-1 and (**E**) FAS (*n* = 3 independent experiments). The scale bar represents 20 μm. Each value represents the mean ± SD (*n* = 9); * *p* < 0.05 versus model; ** *p* < 0.01 versus model.

To better understand the molecular mechanism by which EAOTs mediate the beneficial effects on obese animals described above, two proteins were measured. As shown in the immunoblot analysis, CPT-1, which promotes fatty acid oxidation, was significantly higher in the treatment groups than in the model group (Figure 5C). This was confirmed by immunohistochemistry. The number of CPT-1-positive cells significantly increased with EAOT treatment (Figure 5A).

Moreover, FAS, which enhances fat synthesis, was down-regulated in the EAOT groups, particularly in the 2006 and 1996 groups (Figure 5C). Consistently, the model group revealed intense staining around the lipid inclusions in comparison to the control groups, and EAOTs were able to significantly reduce FAS expression (Figure 5B). All these data indicate that EAOTs might alleviate HFD-induced metabolic abnormalities, at least in part, through regulating the expression of a certain set of metabolic proteins.

### 3.8. EAOTs Inhibit iNOS and TNF-α Expression

**Figure 6:**
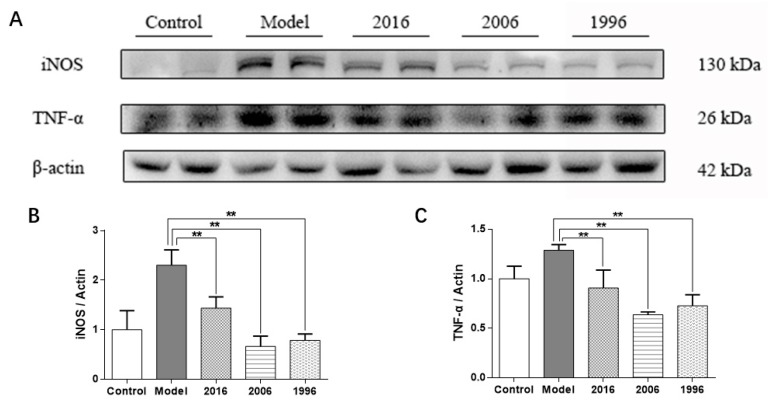
EAOTs inhibit iNOS and TNF-α. (**A**) Western blotting analysis of iNOS and TNF-α protein levels in mice liver and densitometric quantification of (**B**) iNOS and (**C**) TNF-α (*n* = 3 independent experiments). Each value represents the mean ± SD (*n* = 9); ** *p* < 0.01 versus model.

Obesity is usually associated with a large number of inflammatory cells and factors. Severe inflammation can aggravate the liver injury, cause the decline of liver function, and weaken the glucolipid metabolism ability and lipid metabolism [15]. As shown in Figure 6A, the protein expression of iNOS and TNF-α in livers of the model group mice showed a marked increase compared to those of the control group, while the treatment groups had a lower iNOS and TNF-α protein expression level in the liver than those in the model group. Especially in the 2006 group, the expression levels decreased markedly compared to the model group. These results showed that EAOTs can relieve liver inflammation to a certain degree by inhibiting inflammation factors, iNOS and TNF-α.

## 4. Discussion

In the present study, we provided evidence that EAOTs significantly improved obesity phenomena, such as body weight, Lee’s index, fatty liver, adiposity, and hyperlipidemia in mice with HFD-induced obesity. The reduction of body fat accumulation and organ index in the study also indicated that EAOTs modulated obesity in mice. Furthermore, proteins up-regulating fat consumption were higher in the treatment groups, particularly in the 2006 group, and proteins related to fat synthesis and inflammation were down-regulated in the EAOT groups. These results revealed that obesity seemed to be alleviated within six weeks when C57BL/6J mice were given EAOTs daily.

White adipose tissues (WATs) are optimized to store energy in large lipid droplets for later use [16]. The accumulation of body fat in WATs leads to both hypertrophy and hyperplasia of white adipocytes [17]. These changes are associated with obesity-related diseases such as type-2 diabetes and an inflammatory response [18,19]. Previous studies have demonstrated that AMPK is a master energy sensor that integrates nutrients, hormones, and stress signals to maintain whole-body energy homeostasis [20]. Activated AMPKα, namely p-AMPKα, is able to stimulate catabolism and inhibit anabolism, leading to an increase of fat consumption. Rasmus’s study has proved that AMPK in muscle is activated in response to exercise [21]. Moreover, p-AMPK can inactivate ACC1, leading to inhibition of de novo fatty acid and cholesterol synthesis. Phosphorylation of ACC2 by p-AMPK, on the other hand, causes increases of fatty acid oxidation [22]. In addition, the AMPK-ACC pathway is able to suppress the TG production because ACC is the rate-limiting enzyme for the synthesis of malonyl-CoA, which is a critical substrate for fatty acid biosynthesis and a potent inhibitor of fatty acid oxidation [23]. Various phytochemicals used to treat metabolic diseases are often able to stimulate AMPK activation [24,25,26]. In the present study, EAOTs markedly induced AMPKα and ACC phosphorylation in the liver, and increased the p-AMPKα/AMPK and p-ACC/ACC ratio, suggesting that AMPK may be the key mechanism for the beneficial effects of EAOTs on fatty liver.

This speculation was further supported by the observation that EAOTs activated CPT-1, whose expression is inhibited by ACC in the mouse liver. The phosphorylation of ACC promoted by p-AMPKα decreased the activation of Malonyl CoA [27], which is a fatty acid precursor, as well as an inhibitor of CPT-1. Therefore, p-AMPKα promotes the expression of CPT-1 by improving the level of p-ACC and decreasing the expression of Malonyl CoA. CPT-1 is the rate-limiting enzyme of mitochondrial fatty acid oxidation [28], which can increase the capacity of fatty acid flux into the mitochondria. In other studies [29], drugs affect the lipid regulatory system by activating the AMPK-ACC-CPT-1 pathway. Therefore, it is reasonable to speculate that EAOTs relieve disorders of lipid metabolism by activating the AMPK-ACC-CPT-1 pathway.

FAS is a key lipogenic enzyme in mammals, and it catalyzes all the reactions in the conversion of acetyl-CoA and malonyl CoA to palmitate [30]. Its concentration is sensitive to nutritional and hormonal status in lipogenic tissues such as liver and adipose tissues. The nutritional regulation of FAS occurs mainly via changes in FAS gene transcription [31]. Our study has shown that FAS expression is significantly inhibited by EAOT administration, also suggesting that EAOTs’ anti-obesity effects are partly due to the inhibition of expression of fatty acid and TG synthesis-related proteins. However, more experiments are needed to understand the effect of EAOTs on anti-obesity in other body parts.

In addition, it has been reported that AMPK activation led to decreased production of inflammatory mediators in macrophages [32]. In our study, EAOTs stimulated AMPKα phosphorylation and decreased expression of inflammatory factor TNF-α, a potent inflammatory inducer, as well as iNOS. It appears that EAOT-induced AMPK might also contribute to the suppression of inflammatory responses, which would lead to improvements in metabolic disorders.

While the 2006 group showed better anti-obesity and anti-inflammatory effects than the other EAOT treatment groups, it is difficult to speculate which ingredients contributed to this difference, if only relying on the data in this study. As shown in Table 1, we determined the content of free amino acids, total soluble sugar, and tea polyphenols in EAOTs. The free amino acids content was significantly lower in aged oolong tea, and the content of total soluble sugar markedly increased with the storage time. The tea polyphenols content was the highest in 2006 EAOTs, the second highest in 2016 EAOTs, and the lowest in 1996 EAOTs. After correlation analysis, these three ingredients were not related to EAOTs’ anti-obesity and anti-inflammatory effects. It may be the effect of undetermined ingredients, or the combined effect of one known ingredient and other unclear substances, that contributes to these effects. Actually, this question is exactly what we plan to research later. HPLC-MS will be used to determine and analyze more comprehensive ingredients.

In conclusion, EAOT administration has a beneficial effect, reducing body weight gain, adipose tissue weight, and adipocyte size, as well as lipid levels in serum and liver in HFD-induced obese mice. Moreover, EAOTs affect the lipid metabolic regulatory system in the liver by activating the expression of FAS and changing the activation of the AMPK-ACC-CPT-1 pathway, which is known to play a central role in lipid metabolism regulation that facilitates catabolism of fuel storage. In addition, EAOTs could have an impact on inflammation, which protects the liver function and energy metabolism indirectly. Taken together, these results suggest that, as a novel AMPK activator, EAOTs would be a potential candidate for the treatment of obesity and metabolic disorders.

## Figures and Tables

**Table 1:** The ingredients of water extract from aged oolong teas (EAOTs) (g/g).

Sample	Water (%)	Free Amino Acids (%)	Total Soluble Sugar (%)	Tea Polyphenols (%)
2016	0.07 ± 0.01 ^a^	5.13 ± 0.03 ^a^	7.08 ± 0.25 ^a^	39.7 ± 0.3 ^a^
2006	0.08 ± 0.01 ^a^	3.99 ± 0.19 ^b^	8.18 ± 0.08 ^b^	46.5 ± 0.6 ^b^
1996	0.06 ± 0.01 ^a^	3.81 ± 0.04 ^b^	8.39 ± 0.08 ^b^	34.0 ± 0.5 ^c^

The value is the mean ± SD (*n* = 3). Values marked with different lower-case letters in superscript format indicate significant differences; values marked with the same lower-case letters in superscript format indicate no significant differences.

**Table 2:** Organ index of mice fed a HFD and EAOTs for 6 weeks.

Group	Liver/Weight (%)	Kidney/Weight (%)	Epididymal Fat/Weight (%)	Intestinal Fat/Weight (%)	Pararenal Fat/Wright (%)	White Fat/Weight (%)
Control	44.5 ± 0.1 ^a^	13.0 ± 1.0 ^a^	1.30 ± 0.46 ^a^	5.14 ± 2.43 ^a^	4.15 ± 1.98 ^a^	9.31 ± 4.32 ^a^
Model	45.2 ± 0.5 ^a^	9.39 ± 0.88 ^b^	5.35 ± 0.29 ^b^	23.9 ± 5.3 ^b^	30.6 ± 3.3 ^b^	56.3 ± 6.1 ^b^
2016	36.4 ± 0.1 ^b^	11.9 ± 0.9 ^a^	3.02 ± 1.16 ^c^	10.4 ± 5.4 ^a^	16.4 ± 8.0 ^c^	24.7 ± 16.3 ^c^
2006	37.0 ± 0.1 ^b^	12.4 ± 1.5 ^a^	2.77 ± 0.75 ^c^	7.73 ± 3.76 ^a^	11.8 ± 6.1 ^a,c^	16.4 ± 5.6 ^a^^,c^
1996	37.0 ± 0.1 ^b^	12.4 ± 1.6 ^a^	2.91 ± 1.07 ^c^	9.20 ± 4.65 ^a^	12.8 ± 6.5 ^c^	19.7 ± 9.3 ^a^^,c^

The value is the mean ± SD (*n* = 3). Values marked with different lower-case letters in superscript format indicate significant differences; values marked with the same lower-case letters in superscript format indicate no significant differences.

**Table 3:** Serum levels of triglycerides (TGs), total cholesterol (TC), high-density lipoprotein (HDL), and low-density lipoprotein (LDL).

Group	TG (mmol/L)	TC (mmol/L)	HDL-C (mmol/L)	LDL-C (mmol/L)
Control	1.41 ± 0.30 ^a^	4.18 ± 0.58 ^a^	2.78 ± 0.37 ^a^	0.55 ± 0.12 ^a^
Model	1.95 ± 0.34 ^b^	9.25 ± 1.30 ^b^	2.26 ± 0.24 ^a^	1.46 ± 0.33 ^b^
2016	1.45 ± 0.22 ^a^	6.20 ± 0.59 ^c^	4.03 ± 0.44 ^b^	1.00 ± 0.11 ^c^
2006	1.13 ± 0.26 ^a^	5.74 ± 0.46 ^c^	3.80 ± 0.36 ^b^	0.86 ± 0.10 ^c^
1996	1.19 ± 0.24 ^a^	6.42 ± 0.84 ^c^	4.21 ± 0.58 ^b^	0.97 ± 0.24 ^c^

The value is the mean ± SD (*n* = 3). Values marked with different lower-case letters in superscript format indicate significant differences; values marked with the same lower-case letters in superscript format indicate no significant differences.
